# A Simple Gain-Based Evaluation of the Video Head Impulse Test Reliably Detects Normal Vestibulo-Ocular Reflex Indicative of Stroke in Patients With Acute Vestibular Syndrome

**DOI:** 10.3389/fneur.2021.741859

**Published:** 2021-10-29

**Authors:** Björn Machner, Kira Erber, Jin Hee Choi, Andreas Sprenger, Christoph Helmchen, Peter Trillenberg

**Affiliations:** ^1^Department of Neurology, University Hospitals Schleswig-Holstein, Lübeck, Germany; ^2^Department of Anesthesiology and Intensive Care, University Hospitals Schleswig-Holstein, Lübeck, Germany

**Keywords:** stroke, dizziness, vertigo, emergency department (ED), video-HIT

## Abstract

**Objective:** The head impulse test (HIT) assesses the vestibulo-ocular reflex (VOR) and is used to differentiate vestibular neuritis (abnormal VOR) from stroke (normal VOR) in patients presenting with an acute vestibular syndrome (AVS). The video-oculography-based HIT (vHIT) quantifies VOR function and provides information imperceptible for the clinician during clinical bedside HIT. However, the vHIT—like an electrocardiogram—requires experienced interpretation, which is especially difficult in the emergency setting. This calls for a simple, reliable and rater-independent way of analysis.

**Methods:** We retrospectively collected 171 vHITs performed in patients presenting with AVS to our emergency department. Three neuro-otological experts comprehensively assessed the vHITs including interpretability (artifacts), VOR gain (eye/head velocity ratio), velocity profile (abrupt decline) and corrective saccades (overt/covert). Their consensus rating (abnormal/peripheral vs. normal/central) was compared to a simple algorithm that automatically classified the vHITs based on a single VOR gain cutoff (0.7).

**Results:** Inter-rater agreement between experts was high (Fleiss' kappa = 0.74). Five (2.9 %) vHITs were “uninterpretable” according to experts' consensus, 80 (46.8 %) were rated “normal” and 86 (50.3 %) “abnormal”. The algorithm had substantial agreement with the experts' consensus (Cohen's kappa = 0.75). Importantly, it correctly classified all of the normal/central vHITs denoted by the experts (100% specificity) and at the same time it had sufficient sensitivity (75.6%) in detecting abnormal/peripheral vHITs.

**Conclusion:** A simple, automated, gain-based evaluation of the vHIT reliably detects normal/central VOR and may be a feasible and effective tool to screen AVS patients for potentially underlying stroke in the emergency setting.

## Introduction

Patients with acute vestibular syndrome (AVS) suffer from sudden onset of vertigo or dizziness, concurrent nausea/vomiting, gait instability and nystagmus (1). The AVS is most often due to an acute unilateral peripheral vestibulopathy, usually vestibular neuritis (VN), however, up to 25% of AVS are caused by a posterior circulation stroke (PCS) (2–6). The diagnosis of PCS in AVS is challenging for the clinician (7) and even magnetic resonance imaging (MRI) can miss about 20–50% of posterior fossa infarctions in the first 48 h (3, 8). Among the clinical tests and oculomotor signs, that help to differentiate peripheral from central causes of AVS (1), a normal head impulse test (HIT) is the single best predictor for stroke (3). The HIT assesses the function of the vestibulo-ocular reflex (VOR) (9). A bilaterally intact VOR in an AVS patient is a strong indicator of PCS (10). An abnormal VOR (ipsilesionally reduced VOR with subsequent corrective saccade) indicates canal paresis due to a peripheral unilateral vestibulopathy (9). Only rarely PCS can cause a severe VOR deficit leading to an abnormal HIT, that is if the anterior inferior cerebellar artery (AICA) is affected, which also supplies the inner ear (mixed central and peripheral vestibular pathology) (4, 11, 12).

When the HIT is performed as a clinical bedside test, diagnostic accuracy is depending on the experience and skill of the examiner (13–16). Interestingly, novices/non-experts were shown to have higher sensitivity (i.e., detecting abnormal VOR in patients with peripheral vestibulopathy), whereas experts have higher specificity (i.e., detecting a normal VOR in PCS patients) (13). In order to make the HIT more independent of the individual observer and to allow quantitative assessment, video-oculography based HIT systems were introduced (17, 18). The vHIT has higher diagnostic accuracy (sensitivity and specificity) to detect or exclude a VOR deficit than the clinical bedside HIT (19). If performed by experienced staff and interpreted by neuro-otological experts, the vHIT was previously shown to be a very helpful and reliable diagnostic tool in the differential diagnosis (VN vs. PCS) of AVS patients, leading to the term “ECG for the eyes” in analogy to the electrocardiography in acute chest pain patients (14).

Nonetheless, to our knowledge, the vHIT is not yet widely established in clinical emergency settings. This may be due to different reasons: (i) Technical performance and acquisition of the vHIT must be trained and can still be influenced by artifacts (especially poor calibration, impaired pupil detection, low head velocity or goggle slippage during head thrusts) which may influence the test outcome (20–22). (ii) The vHIT is quantified by calculating the VOR gain, which basically refers to the ratio of eye and head velocity. However, the different commercially available vHIT devices use different ways of calculating the VOR gain (23), making it difficult to define one cutoff. Hitherto, there is no final consensus on an absolute gain value below which one considers the VOR gain as pathological (24). Proposed cutoffs are 0.68 (24), 0.7 (16, 19, 25) or 0.8 (22, 26, 27). (iii) Basing the final judgement (normal vs. abnormal) of the vHIT solely on the VOR gain may not be sufficiently reliable. It ignores other indicators of a deficient VOR (e.g., abrupt decline of eye velocity profile, overt/covert corrective saccades, anti-compensatory “wrong-way” saccades) (20, 28–30). These “subtle” signs of an abnormal/peripheral VOR are usually not analyzed by commercially available vHIT systems and can only be recognized by the eyes of an experienced observer or additional software tools (31).

Hence, there is clearly the need for a simple but at the same time reliable way of vHIT interpretation to increase its use and usability for clinical decision-making in the emergency setting. By retrospective analysis of vHIT data originally obtained from patients presenting with AVS to our emergency department (6), we investigated whether a simple algorithm based on a single VOR gain cutoff value (0.7) would be as good as neuro-otological experts, who could comprehensively assess the complete vHIT trace, in classifying a VOR as normal (indicating central etiology of AVS, e.g., stroke) or abnormal (pointing to peripheral vestibulopathy).

## Methods

### Study Design, Setting and Population

We first searched our in-house register of over 600 dizzy patients, that was originally compiled by reviewing medical charts of adults who presented with dizziness, vertigo or imbalance to the emergency department (ED) at the University Medical Center in Lübeck/Germany (6), for those who presented with AVS, i.e., an acute onset of dizziness/vertigo within the last 72 h, symptoms still persistent at presentation in the ED and presence of a spontaneous nystagmus on clinical examination. Next, we excluded those who had not received a vHIT during the further stay in the hospital.

We also extracted the clinical diagnosis of each patient from the medical charts, which was made based on clinical assessment, course of symptoms and additional diagnostic studies (e.g., brain imaging). In those with imaging-confirmed stroke, the localization of the stroke lesion was collected from the official neuroradiological report.

The study was approved by the Ethics Committee of the University of Lübeck (18-146A) and has been performed in accordance with the ethical standards laid down in the 1964 Declaration of Helsinki and its later amendments. Due to the retrospective design and use of anonymized data, individual written informed consent was not required.

### Video-Oculography Device, Acquisition of vHIT and Automated Gain Calculation

The vHIT was recorded during clinical routine using the EyeSeeCam® HIT System (Autronics, Hamburg, Germany). The patient was seated on a chair and fixated a LED target at a distance of 100 cm. After calibration, a medical-technical assistant standing behind the patient delivered repetitive passive and rapid head rotations (peak velocity: 200–250°/s, amplitude: 10–15°) in the plane of the horizontal semicircular canals. HITs were unpredictable for direction and onset. The device records head and eye velocity traces. Grossly invalid HIT trials are immediately rejected by the device's software. After the recording, the device automatically calculates and plots the VOR gain for each HIT trial and provides an average VOR gain value for each side at a time interval of 40, 60, and 80 ms after HIT onset. For further analysis, we used the patient's mean VOR gain at 60 ms on each side, as previously suggested (17, 23, 32).

### Standardized Assessment of vHIT Traces by Neuro-Otological Experts

All vHITs were offline and independently reassessed by three neuro-otological experts (BM, PT, CH) following a standardized protocol. The raters were blind for the patients' history and examination findings at the time of vHIT assessment. They were provided with the patients' VOR gain at 60 ms on the left and on the right. For each patient, the experts visually inspected the vHIT traces on either side and assessed them for the following variables (20): (i) interpretability (vHIT “interpretable” or “uninterpretable” due to severe disruptive artifacts), (ii) slow eye movements' velocity profile (“normal/bell-shaped”, “abrupt decline”), (iii) fast phase eye-movements: “covert/overt corrective saccades”, “anti-compensatory quick eye movements (AQEM, “wrong-way saccades”) and “unspecific saccades”, (iv) final judgement (normal or abnormal VOR).

### Outcome Measures

Based on the automatically calculated bilateral VOR gains, one “algorithm-based vHIT result” (abnormal or normal) was obtained for each patient. To this end, the VOR was classified as ‘abnormal’ if the gain of at least one side was equal or below 0.7 and as “normal” if VOR gain values on both sides were above this threshold. This cutoff was chosen as it is well below the reported normative values of VOR gains in healthy subjects (18, 24, 26) and it was shown to distinguish well between PCS and VN in smaller cohort studies of AVS patients (14, 16, 25).

From each expert, the side-specific ratings were aggregated to achieve one VOR result (normal, abnormal or uninterpretable) for each patient. The VOR result was classified as “normal”, if the VOR on both sides was rated as normal. The VOR result was “abnormal”, if the VOR on at least one side was rated as abnormal. Furthermore, the VOR result could be classified as “uninterpretable”, if the vHIT on at least one side was rated as uninterpretable due to artifacts.

Based on the vHIT ratings of the three experts, the inter-rater agreement was assessed by calculating the Fleiss' kappa (33). Next, one “expert consensus” was obtained for each patient's vHIT by taking the VOR result that at least two experts agreed on. After removal of the uninterpretable vHITs, the congruency between “experts” consensus' and “algorithm's judgement” was assessed by calculating Cohen's kappa (34).

Finally, we sought to investigate any discrepancies between experts' vHIT judgements and the algorithm by analyzing single variables like “corrective saccades” or the “eye velocity profile” in more detail.

### Statistical Analysis

Statistical analyses were performed using SPSS 26 (IBM Corp., Somer/NY, US). Descriptive statistics were calculated for variables of interest, data are presented as counts and percentages. Inter-rater reliability was calculated using Cohen's kappa for two and Fleiss' kappa for more than two raters (see previous section). The significance level was set at *p* < 0.05.

## Results

The vHITs of 171 AVS patients [mean age 64 years, range 24–95; *n* = 82 (48%) female] were included in the analysis. Regarding their clinical diagnosis, 85 of 171 patients had a peripheral vestibulopathy (usually vestibular neuritis), 37 patients had a diagnosis affecting the central nervous system [*n* = 24 ischemic stroke, *n* = 10 transient ischemic attack (TIA), *n* = 3 inflammatory demyelinating disease] and in 49 patients the diagnosis remained unclear, including those with a remission of symptoms and unremarkable diagnostics. In 15 of 24 patients with a diagnosis of ischemic stroke, the lesion was confirmed by brain imaging (computed tomography and/or MRI). The localization of the stroke lesion was in n=8 the territory of the posterior inferior cerebellar artery (PICA), in *n* = 2 the superior cerebellar artery (SCA) and in *n* = 5 the ponto-/medullary brainstem. There was no patient with an infarction of the AICA in this cohort.

The vHITs were performed at a median time point of 1 day after the patient's admission to the hospital (95% CI: 0–4 days).

**Figure 1:**
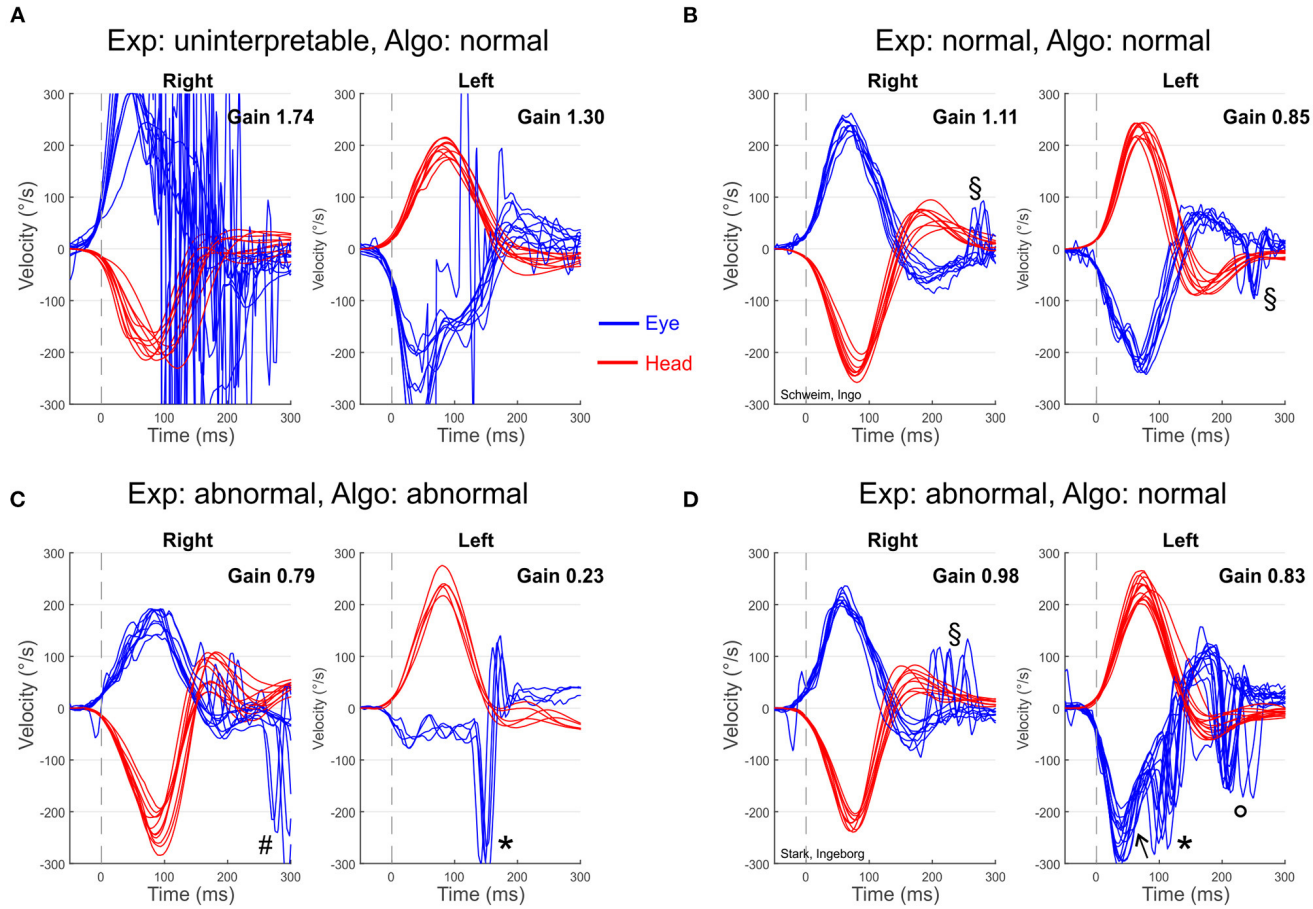
Exemplary vHIT plots of individual patients presenting with AVS including the corresponding judgement by the experts (Exp) and the algorithm (Algo). **(A)** Severe artifacts (noise probably due to impaired pupil detection) and a bilateral implausible high gain (probably due to poor calibration and goggle slippage) make the vHIT of a 94-year-old women (patient ID 143) uninterpretable (experts' judgment). The algorithm classifies it as “normal” because VOR gains are above the cutoff value of 0.7. **(B)** 81-year-old man (ID 169) with ischemic stroke in the territory of right posterior inferior cerebellar artery. Both the experts and the algorithm judged this vHIT as normal/central. **(C)** 75-year-old man (ID 30) with vestibular neuritis on the left side (MRI normal). Both the experts and the algorithm rated his vHIT as abnormal/peripheral. **(D)** 80-year-old woman (ID 84) with vestibular neuritis on the left side (MRI normal). The experts used additional information apart from the VOR gain (abrupt decline of eye velocity, overt/covert corrective saccades) to state a “abnormal/peripheral” VOR, while the algorithm rated this vHIT as normal/central according to the bilateral VOR gain above 0.7. *covert corrective saccade; °overt corrective saccade; ^#^anti-compensatory quick eye movement (AQEM, “wrong-way” saccade toward deficient VOR); ^↖^Abrupt decline of eye velocity profile; ^§^small unspecific saccades.

The inter-rater reliability between the three experts was high (Fleiss' kappa = 0.743, SEM 0.039, *p* < 0.001). In 135 of 171 patients (78.9 %), there was absolute agreement in the rating of the vHIT between all three experts. To obtain a unified expert rating of each vHIT (experts' consensus) for further analyses, the judgement of every vHIT was used that at least two experts agreed on. Experts' consensus rated the vHIT in five patients (2.9%) as “uninterpretable” due to artifacts (see Figure 1A for an example). The vHITs of 80 (46.8 %) patients were found to be “normal” and those of the remaining 86 (50.3 %) were judged as “abnormal” by the experts. The uninterpretable vHITs were excluded from the upcoming analyses.

The algorithm's vHIT judgements were highly congruent with the experts' consensus (Table 1). This was confirmed by a substantial inter-rater agreement: Cohen's kappa = 0.75 (SEM 0.05, *p* < 0.001). Particularly, all of the vHITs that were rated as normal by experts' consensus were also correctly classified as normal by the algorithm (100% “true negatives”; Table 1, see Figure 1B for an example). Hence, the number of “false positives” was 0. Furthermore, the algorithm correctly identified 65 of the 86 expert-classified pathological vHITs as abnormal (75.6 % “true positives”; Figure 1C).

**Table 1:** Cross table of the algorithm's (VOR gain cutoff at 0.7) and experts' judgements on the vHITs of 166 patients with AVS.

***vHIT rating***		**Algorithm**		
		**Normal**	**Abnormal**	**Total**
**Experts**	**Normal**	80 (48.2)	0 (0)	80 (48.2)
	**Abnormal**	21 (12.7)	65 (39.1)	86 (51.8)
	**Total**	101 (60.9)	65 (39.1)	166 (100)

*Data are n (%)*.

However, the algorithm falsely classified the vHITs of 21 patients as normal that were all judged as abnormal by the experts (12.7% “false negatives”; Table 1). All these patients had bilateral VOR gain values above 0.7 and were therefore classified as “normal” by the algorithm. However, experts found additional indicators of deficient VOR function (Figure 1D): in 19 of 21 (91%) patients the eye velocity profile exhibited an abrupt decline, in 17 (81%) there were covert and in 21 (100 %) overt corrective saccades.

Furthermore, in an additional analysis (Table 2) we investigated the performance of the algorithm if the gain cutoff for an abnormal VOR was set at 0.8 instead of 0.7. Applying the higher cutoff led to an increase in the algorithm's sensitivity (88.4% vs. 75.6%) as it detected more abnormal (peripheral) vHIT results (76 of 86), but at the same time there was a decrease in specificity (90% vs. 100%) because 8 of 80 normal (central) vHITs were now classified as abnormal/peripheral.

**Table 2:** Cross table of algorithm's and experts' vHIT judgements with a modified VOR gain cutoff at 0.8.

***vHIT rating***		**Algorithm**		
		**Normal**	**Abnormal**	**Total**
**Experts**	**Normal**	72 (43.4)	8 (4.8)	80 (48.2)
	**Abnormal**	10 (6.0)	76 (45.8)	86 (51.8)
	**Total**	82 (49.4)	84 (50.6)	166 (100)

*Data are n (%)*.

Finally, we specifically investigated the performance of the original algorithm (gain cutoff at 0.7) in patients with the clinical diagnosis of ischemic stroke. All of them (*n* = 24) had a bilateral VOR gain above 0.7 and were correctly classified as “central” by the algorithm (as well as by the experts).

## Discussion

By retrospective investigation of vHITs obtained in a considerable sample of AVS patients (*n* = 171), we could show that a simple gain-based algorithm is as good as human expert observers in detecting normal VOR function. This is clinically relevant, because a normal VOR in an AVS patient is the single best predictor for a stroke (3). Studies with smaller sample sizes but detailed clinical information of AVS patients (including MRI results and clinical follow-up) previously showed that a VOR gain of 0.7 is an excellent cutoff value to discriminate stroke (bilateral VOR gain above threshold) and VN (VOR gain on one side equal/below threshold) (14, 16, 25). Our study adds that an automated, rater-independent, purely gain-based assessment is as good as the human rating by expert observers in detecting normal (central) vHIT results. In our cohort (no AICA strokes), the algorithm (gain cutoff 0.7) correctly classified all patients with ischemic stroke as “central”. Others have suggested higher gain values as a cutoff for pathological VOR (e.g., 0.8) (11, 22, 27). As shown by our additional analysis, a higher cutoff (0.8) can indeed increase the algorithm's sensitivity for peripheral vestibulopathy but at the same time reduces its specificity, thereby increasing the risk to falsely classify a stroke patient's VOR as abnormal/peripheral. Especially when evaluating AVS patients in an emergency setting, identification of stroke is most critical for clinical decision-making on diagnostics, monitoring and therapy (35). For this population and clinical setting, we [and others (25)] suggest to use the 0.7 as a more conservative cutoff gain for pathological VOR. Of course, such a strict cutoff bears the risk to falsely classify some VN patients with borderline gain values (e.g., 0.71–0.8) as “stroke”. However, in the emergency setting, we regard such a “false-serious” misdiagnosis less critical than a false-benign VN diagnosis in a stroke patient. Furthermore, there are also stroke patients (usually cerebellar/pontine lesions) revealing a mild (usually bilateral) VOR gain reduction that would be falsely classified when applying a more lenient gain cutoff of 0.8 or higher (11). In the acute evaluation of AVS patients it appears principally acceptable to use a rather simple but very specific gain-based algorithm that ignores other indicators of VOR dysfunction and thereby missing some VN diagnoses. In our cohort, more than 75% of patients with an abnormal/peripheral finding on the vHIT, as judged by human experts, were still correctly classified by the automated gain-based algorithm, which is reasonable. As expected, expert raters make also use of other markers of VOR dysfunctions, such as the abrupt decline of the eye velocity profile or presence of corrective saccades, to make their decision. However, it must be doubted that non-expert raters in the ED could equally correctly interpret all the possible fast phase eye movements on the vHIT, including corrective saccades, CAQEMs but also blinks, artifacts and unspecific small saccades. Furthermore, the commercially available vHIT devices do not provide a systematic analysis of corrective saccades and hitherto, there are only custom-made software tools available for rather experienced users or research purposes (31, 36). Therefore, it appears more feasible and reliable to apply a purely gain-based approach of vHIT analysis that might be slightly less sensitive for abnormal/peripheral VOR findings (indicative of VN) but very specific for normal/central results pointing to a PCS.

Alternatively, one could argue that the clinical bHIT is already sufficient to identify those AVS patients with normal/central VOR. This may indeed be the case if experienced neuro-otologist perform and interpret the bHIT (1). However, we know from different studies that the bHIT's specificity (percentage of stroke patients correctly detected) is markedly reduced (to about 64%) when applied by non-experts (13, 16). Therefore, in an emergency setting, where a neuro-otology expert may not always be available, a rater-independent vHIT algorithm may be superior to the clinical bHIT assessment by non-experts (14).

### Study Limitations and Potential Pitfalls of Gain-Based vHIT Analysis

The aim of this study was to assess whether a purely gain-based algorithm performs as good as human expert observers in differentiating normal and abnormal VOR function on the vHIT of AVS patients. As opposed to previous studies on clinical and/or video-based HITs in smaller cohorts of AVS patients (16, 25), due to the retrospective character of the study, our sub-analysis of patients with ischemic stroke was based on the clinical diagnosis derived from the medical charts, which was sometimes not confirmed by MR brain imaging. This might imply the risk of some misdiagnoses and false conclusions.

Furthermore, the vHIT recordings, that were used to assess the different evaluation methods (experts vs. algorithm), were acquired by experienced staff during clinical routine. We cannot exclude that the accuracy of gain-based vHIT evaluation would drop if the vHIT is recorded by a less experienced investigator. Hence, a training to correctly perform the head thrust and record the vHIT is inevitable before using the device in any setting, including the ED. Fortunately, staff can be relatively quickly trained and learning curves are usually steep (21). Nevertheless, while the software of HIT devices usually excludes gross artifacts that occur during the head thrust, different other confounders are not automatically detected and removed. These include errors during calibration (false high gain values), a slippage of the goggles, too low head velocity and noisy eye signal due to pupil loss or mascara, which all may not be detected by the device's software but may severely influence the gain (21, 22, 37). An experienced human observer has the advantage to visually check for these artifacts and assess the overall interpretability of the vHIT plot before relying on the VOR gain. Such a first check for massive artifacts and overall interpretability through a human observer must never be skipped and cannot be replaced by an automated software tool by now. Fortunately, a previous study could show that single eye traces that are disturbed by artifacts do not have a significant impact on mean VOR gain calculation and therefore do not reduce the test's accuracy or challenge the chosen gain cutoff of 0.7 (37).

Our study was based on VOR gain values (instantaneous gain at 60 ms) calculated by the software of the EyeSeeCam® vHIT device. However, the different commercially available vHIT devices use different ways of calculating the VOR gain (23), which may result in different VOR gain results and challenge one VOR gain value as cutoff for pathology. Nonetheless, we propose that our suggested VOR gain cutoff can be translated to other systems, for two reasons: First, the gain calculation approach of the two most widely used vHIT systems, i.e., area under the curve (Otometrics ICS Impulse®) or instantaneous gain at a defined time interval (EyeSeeCam® vHIT), yield very similar gain results (particularly, when assessing the affected ear) and are very consistent in the overall classification (normal/abnormal) of the vHIT result (23). Second, the same VOR gain cutoff of 0.7 was successfully applied in a previous study by Mantokoudis et al., who used the Otometrics ICS Impulse® to record and analyse vHITs in AVS patients to discriminate VN and PCS (25).

## Conclusions

**Figure 2:**
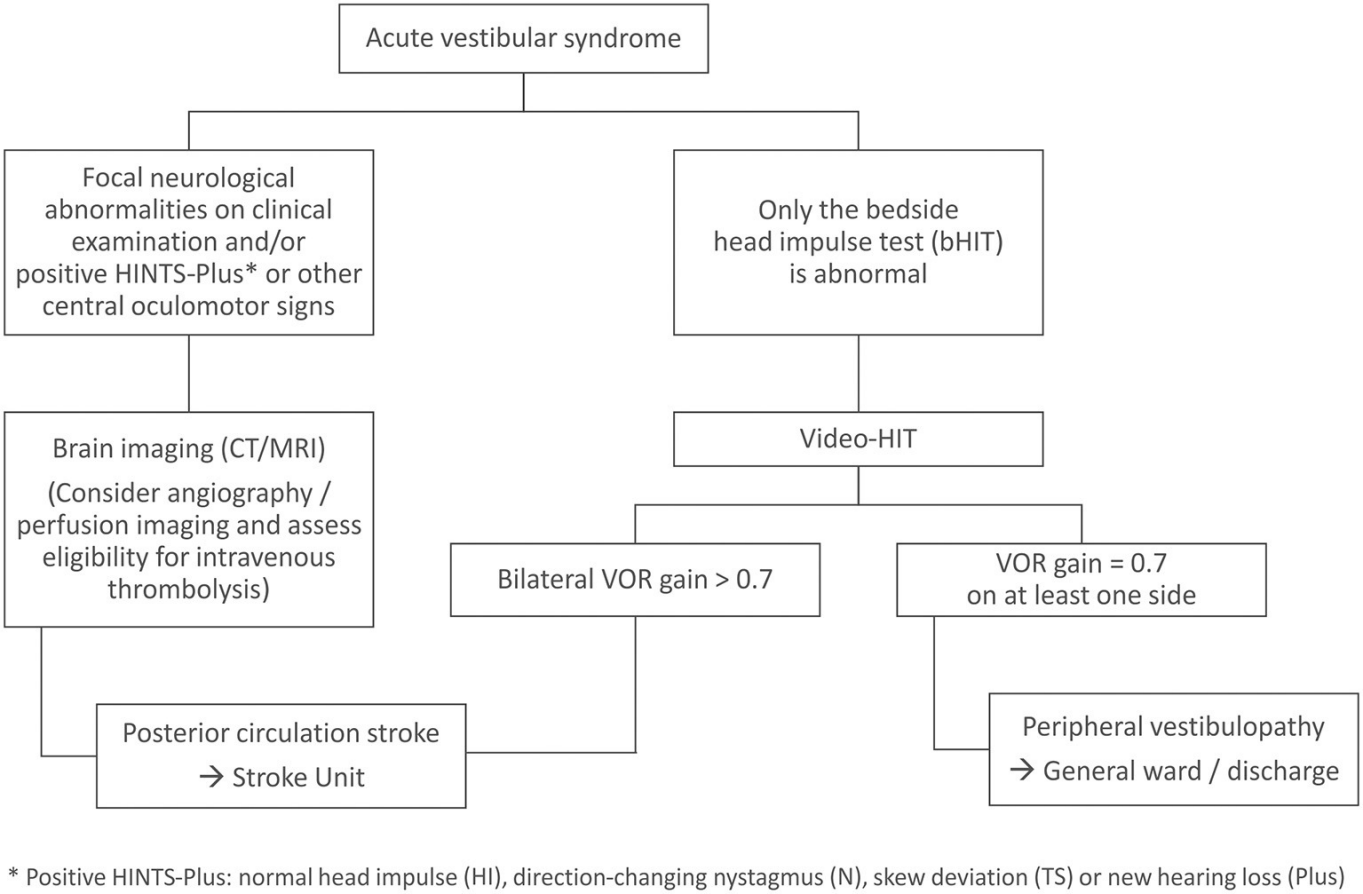
Clinical pathway for patients presenting with AVS to the emergency department.

Based on the findings of the current and previous studies (14, 16, 25), we suggest that performing the vHIT by trained staff and evaluating it with a simple gain-based algorithm may be a feasible and reliable tool to improve the diagnosis and management of AVS patients in the emergency setting. As long as the two clinical trials (NCT02483429 by Newman-Toker and collaborators; U1111-1172-8719 by Mohwald et al. (38)), which prospectively assess the usability of vHIT devices in the management of AVS patients in the ED, are still under investigation, we suggest the following clinical pathway based on current knowledge (Figure 2). Patients presenting with isolated AVS (i.e., no focal neurological abnormality indicating central nervous system involvement) should first be clinically investigated for central oculomotor signs including the HINTS (Head Impulse, Nystagmus and Test-of-Skew), as these are very sensitive for a central etiology of the AVS, usually ischemic stroke of the posterior circulation (1). Furthermore, patients should also be screened for presence of a new hearing loss (HINTS *Plus*), as this points to an AICA stroke with combined infarction of the inner ear (3, 39, 40). If only the clinical HIT result is abnormal (16), this should be verified by additional vHIT recording. If the VOR gain of at least one side is equal or below 0.7, patients should be stratified to the suspected diagnosis of peripheral vestibulopathy and not undergo further stroke-related diagnostics, as this can save imaging and monitoring resources or even unnecessary hospitalization. In contrast, those with a bilateral VOR gain above 0.7 should be assigned to “suspected PCS” and accordingly receive the necessary stroke assessment including brain imaging, monitoring on a stroke unit and where appropriate antiplatelet/anticoagulatory medication, and in severe cases should also be evaluated for potential eligibility for intravenous thrombolysis (35).

## Data Availability Statement

The raw data supporting the conclusions of this article will be made available by the authors, upon reasonable request.

## Ethics Statement

The study was approved by the Ethics Committee of the University of Lübeck (18-146A) and has been performed in accordance with the ethical standards laid down in the 1964 Declaration of Helsinki and its later amendments. Written informed consent for participation was not required for this study in accordance with the national legislation and the institutional requirements.

## Author Contributions

BM: conceptualization, methodology, investigation, data analysis, visualization, and writing—original draft. KE: investigation and data analysis. JC: investigation, data analysis, and project administration. AS: investigation, visualization, and software. CH: writing—review & editing and supervision. PT: conceptualization, methodology, writing—review & editing, and supervision. All authors contributed to the article and approved the submitted version.

## Funding

This work was supported by the German Research Foundation (Deutsche Forschungsgemeinschaft; Grant MA5332/3-1 to BM).

## Conflict of Interest

The authors declare that the research was conducted in the absence of any commercial or financial relationships that could be construed as a potential conflict of interest.

## Publisher's Note

All claims expressed in this article are solely those of the authors and do not necessarily represent those of their affiliated organizations, or those of the publisher, the editors and the reviewers. Any product that may be evaluated in this article, or claim that may be made by its manufacturer, is not guaranteed or endorsed by the publisher.
